# Research progress in neuroimaging of sporadic early-onset Alzheimer’s disease

**DOI:** 10.3389/fneur.2026.1788814

**Published:** 2026-04-10

**Authors:** Xiang Zhang, Chao Yang

**Affiliations:** The First Affiliated Hospital of Dalian Medical University, Dalian, China

**Keywords:** early-onset Alzheimer’s disease, functional connectivity, logopenic variant of primary progressive aphasia, multimodal imaging, network-based neurodegeneration, posterior cortical atrophy

## Abstract

Early-onset Alzheimer’s disease (EOAD) is defined as Alzheimer’s disease (AD) with an age at onset younger than 65 years, accounting for approximately 5% of all AD cases. More than 90% of EOAD cases do not carry autosomal dominant pathogenic mutations. Although its prevalence is lower than that of late-onset Alzheimer’s disease (LOAD), EOAD follows a more aggressive clinical course. A subset of EOAD patients present with non-amnestic variant phenotypes, including logopenic variant of primary progressive aphasia (lvPPA), frontal variant Alzheimer’s disease (fvAD), posterior cortical atrophy (PCA), and corticobasal syndrome (CBS). However, the neuroimaging characteristics of EOAD and their differences from those of LOAD remain poorly elucidated to date. Therefore, this review systematically summarizes the recent research progress in neuroimaging of EOAD, including structural, functional, and metabolic imaging modalities. We also discuss the potential pathogenesis of EOAD, with the aim to provide evidence-based reference for the development of EOAD-specific imaging assessment systems and the optimization of disease efficacy monitoring protocols in future research.

## 1 Introduction

Alzheimer’s disease (AD) is a neurodegenerative disorder defined by core neuropathological hallmarks: amyloid-*β* (Aβ) plaques and neurofibrillary tangles composed of hyperphosphorylated tau protein, and it represents the most common cause of dementia worldwide. AD with symptom onset after the age of 65 years is classified as late-onset Alzheimer’s disease (LOAD), which is the typical form of the disease. In contrast, approximately 5% of AD patients develop clinical symptoms before the age of 65 years, which is the conventional threshold for the definition of early-onset Alzheimer’s disease (EOAD) in the literature (1, 2). Epidemiological data show that the number of global EOAD cases among adults aged 40–64 years more than doubled between 1990 and 2021, with substantial increases in its prevalence, incidence, and mortality (3).

Approximately 5–10% of EOAD patients carry autosomal dominant mutations (in *APP, PSEN1*, or *PSEN2*) that drive early cerebral Aβ aggregation, while more than 90% of EOAD cases are sporadic. The diagnosis of sporadic EOAD is delayed by approximately 1.6 years compared with that of elderly AD patients, making this population a major challenge in AD diagnosis and management (4). EOAD patients typically exhibit more prominent impairments in executive function, language, visuospatial processing, and other cognitive domains (5–9), which correlate with neurodegeneration in the posterolateral temporal lobe, posteromedial parietal lobe, frontal lobe, or occipital cortex. Neuropathological studies have also confirmed a “hippocampal-sparing” subtype of AD, which is predominantly observed in younger patients (10). Furthermore, compared with LOAD patients, EOAD patients have a more aggressive disease course, characterized by faster rates of cerebral atrophy, lower Mini-Mental State Examination (MMSE) scores, and more rapid cognitive decline (5, 11, 12). Existing studies suggest that higher tau burden and more severe neuroinflammatory responses in EOAD may underlie its aggressive clinical features (12–20).

Current AD research and clinical management frameworks are centered on typical LOAD. Reliable fluid and imaging biomarkers for LOAD have been fully validated, and artificial intelligence-assisted diagnostic technologies can effectively predict the pathological and clinical progression of individuals with mild cognitive impairment (MCI) or cognitively normal (CN) status (21–24). However, due to the unique age at onset and clinical phenotypic heterogeneity of sporadic EOAD, this population is usually excluded from large-scale observational and therapeutic clinical trials. This exclusion directly leads to delayed diagnosis, calibration bias of existing biomarkers in this population, and inability to extrapolate clinical trial results to sporadic EOAD patients. Ultimately, the pathological characteristics, disease progression patterns, and standardized diagnosis and treatment protocols for sporadic EOAD have not been systematically elucidated (25).

Neuroimaging techniques play an irreplaceable core role in EOAD research, for four key reasons: First, they enable *in vivo* visualization of abnormal alterations in cerebral structure, function, and molecular pathology, compensating for the scarcity of autopsy neuropathological data on sporadic EOAD. Second, they provide quantifiable and reproducible biomarkers of disease progression, overcoming the subjectivity and lag of clinical cognitive rating scales. Third, they can serve as *in vivo* visualization tools for targeted therapeutic targets and efficacy monitoring. Based on the above background, this paper systematically reviews the recent research progress in structural MRI, functional MRI, and PET imaging of sporadic EOAD. We aim to (i) focus on the neuroimaging characteristics of EOAD and their differences from those of LOAD; (ii) explore the potential pathophysiological mechanisms of EOAD in combination with existing imaging findings; and (iii) critically evaluate the limitations of current research and future research directions.

## 2 Methodology

This article is a narrative review. Literature retrieval was performed primarily based on the PubMed database, covering the publication period from 2010 to 2025. The search keywords included “early-onset Alzheimer’s disease,” “sporadic early-onset Alzheimer’s disease,” “logopenic variant of primary progressive aphasia “, “posterior cortical atrophy “, “MRI,” “BOLD,” “DTI,” “ASL,” “amyloid PET,” “tau PET,” “FDG PET,” “TSPO PET,” and “SV2A PET”.

The inclusion criteria were as follows: (i) study subjects were patients with sporadic EOAD, including comparative analyses between EOAD and cognitively normal (CN) controls, early-onset non-AD dementia (EOnonAD), and LOAD; (ii) the core content of the study focused on neuroimaging (MRI/PET) biomarkers and their associations with fluid biomarkers; (iii) full-text original research (cross-sectional/longitudinal studies), high-quality systematic reviews, and meta-analyses published in English. Studies with larger sample sizes and standardized image acquisition and analysis methods were preferentially included, and studies on EOAD with different clinical phenotypes were also included to reflect the heterogeneity of the disease. Due to the overall limited sample size of EOAD studies, strict methodological quality scoring of the literature was not performed, but the methodological limitations of each study were addressed in the discussion section.

## 3 Magnetic resonance imaging (MRI)

MRI techniques enable non-invasive assessment of neurodegenerative changes in EOAD across multiple dimensions, including cortical thickness, white matter microstructure, brain functional networks, and cerebral perfusion. It is currently the most widely used imaging modality in clinical EOAD research.

### 3.1 Structural MRI

Structural MRI studies have consistently demonstrated cortical atrophy in EOAD patients in the inferior parietal lobule, superior parietal lobule, precuneus, middle temporal gyrus, inferior temporal gyrus, posterior cingulate gyrus, middle frontal gyrus, superior frontal gyrus, and fusiform gyrus. Notably, this characteristic cortical atrophy pattern across these 9 brain regions was not observed in EOnonAD group, indicating its specificity for differentiating EOAD from other early-onset cognitive impairment disorders (26, 27). Network-based characteristic gray matter atrophy patterns have also been observed in lvPPA (28–30) and PCA (28, 31, 32) (the language network in lvPPA and the visual network in PCA). Furthermore, compared with LOAD, EOAD patients exhibit significantly more severe atrophy in the inferior parietal lobule and precuneus. Based on findings from the Chinese Aging and Neurodegenerative Disease Initiative (CANDI) cohort, precuneus volume in the EOAD group was significantly negatively correlated with serum glial fibrillary acidic protein (GFAP) levels, while no such association was observed in the LOAD group (33).

However, there remains controversy regarding the laterality of the atrophy pattern in EOAD. Previous studies have reported lateralized atrophic changes in EOAD, particularly in the left temporal and parietal lobes (34), as well as interhemispheric differences in functional network connectivity (35). This discrepancy in laterality findings may be attributed to differences in the distribution of clinical phenotypes in the enrolled samples or variations in statistical power caused by different sample sizes across studies.

Most of the above studies are cross-sectional designs with limited sample sizes and single ethnic populations. Future studies need to perform longitudinal follow-up of EOAD patients stratified by clinical phenotype in larger cohorts, and analyze the associations between sensitive fluid biomarkers and regional brain alterations. Such research may improve the clinical applicability of the characteristic atrophy patterns of EOAD, to establish a more accurate diagnostic and subtyping system for the disease.

#### 3.1.1 T2-weighted imaging

White matter hyperintensities (WMHs) appear as regions of relatively high signal intensity on T2-weighted images.

A study based on the Longitudinal Early-onset Alzheimer’s Disease Study (LEADS) cohort found that the spatial distribution of WMHs across regions in the left and right cerebral hemispheres was similar in the EOAD group, with the highest WMH volume in the frontal and parietal regions and the lowest WMH volume in the temporal lobe. Compared with the CN and EOnonAD groups, the EOAD group had a significantly higher mean WMH volume, with the most prominent differences observed in the frontal, parietal, and occipital regions. In addition, total WMH volume (sum across all regions) was significantly correlated with tau burden in the EOAD group (36). The regional distribution of subcortical WMHs differed between lvPPA and PCA, with these regional variations largely aligning with the regional patterns of neurodegeneration associated with these two syndromes (37, 38). Both lvPPA and PCA had greater subcortical and periventricular WMHs in the occipital lobe compared with the age-matched amnestic AD group.

Luo et al. investigated the differences in CSVD injury patterns between EOAD and LOAD patients and their impact on cognitive impairment using the peak width of skeletonized mean diffusivity (PSMD) (39). Compared with traditional CSVD markers such as WMHs, PSMD values showed the strongest correlation with most cognitive domains. However, the specificity of PSMD values in EOAD requires further validation.

It remains unclear whether increased WMH volume in AD is a manifestation of comorbid cerebral small vessel disease (CSVD) or a consequence of AD-related pathological processes. The mechanisms driving WMH are likely complex and involve an interplay between age-related vulnerability and neurodegeneration. Further studies are needed to investigate underlying mechanisms.

#### 3.1.2 Diffusion tensor imaging (DTI)

DTI can visualize the anisotropy and structural integrity of white matter fiber tracts, and is highly sensitive to early microstructural damage in the cerebral white matter.

The comparability of results from existing DTI studies is limited by differences in regions of interest, voxel-based analysis, or tract-based spatial statistics methods used across studies. However, most studies consistently found that, compared with healthy controls, EOAD patients showed reduced fractional anisotropy (FA) in extensive white matter regions (posterior thalamus, genu of the corpus callosum) and increased mean diffusivity (MD) in posterior white matter regions (parietal and occipital lobes) on DTI (40). DTI analyses consistently reveal that lvPPA is characterized by bilateral, yet predominantly left-sided, microstructural alterations within frontally originating white matter pathways (including the superior and inferior longitudinal fasciculi and the uncinate fasciculus) as well as the parietotemporal junction (41–43), whereas PCA demonstrates predominantly right-sided white matter microstructural changes involving the superior and inferior longitudinal fasciculi, inferior fronto-occipital fasciculus, and right fronto-parietal pathways (44, 45). These impairments involve both long-range deep fiber tracts and short-range superficial fibers, disrupting the central hubs of information transmission between brain regions (46, 47). Meanwhile, compared with LOAD, white matter damage in EOAD patients is predominantly located in the posterior cerebral white matter (posterior cingulate gyrus and parietal lobe) and major fronto-parietal white matter pathways, with relative sparing of the medial temporal lobe (47).

Notably, regardless of the clinical phenotype of EOAD, patients exhibit more extensive white matter damage than would be predicted by the degree of gray matter atrophy, suggesting that white matter damage may be an early pathological change in EOAD that precedes cortical atrophy (48). However, this conclusion requires further validation in longitudinal studies.

### 3.2 Resting-state fMRI (rs-fMRI)

rs-fMRI assesses the strength of functional connectivity between brain regions by detecting low-frequency fluctuations in the blood oxygen level-dependent (BOLD) signal, providing an important perspective for investigating the early pathological mechanisms of the disease.

Existing cross-sectional studies have shown that EOAD is mainly characterized by reduced connectivity in the fronto-parietal networks (including the executive control network, salience network, language network, and high-order visual network) (35, 42, 49–53), rather than the reduced medial temporal lobe-hippocampal connectivity typical of LOAD (54–57). Longitudinal studies have demonstrated that brain regions with strong functional connectivity in EOAD have higher baseline tau-PET uptake and more rapid tau-PET accumulation over time. Functional connectivity of the core tau deposition regions can effectively predict the spreading pathway of tau protein. This association between connectivity and tau progression is highly consistent across EOAD patients with different clinical phenotypes (58).

In addition, although EOAD with different clinical phenotypes may involve distinct neural functional networks, tau deposition is consistently observed in the posterior nodes of the DMN (59, 60), and baseline tau burden in the DMN has important predictive value for cognitive decline in EOAD patients (61). However, differences in functional network parcellation methods, seed point selection, and connectivity definitions across studies mean that the reproducibility of these results remains to be verified.

Based on the network-based neurodegeneration theory, pathological proteins spread through the brain via network connections of the cerebral cortex in typical AD (62–65). Accordingly, the study hypothesized that these differences in network connectivity are likely related to the distinct distribution patterns of pathological proteins between EOAD and LOAD.

Taken together, further longitudinal studies tracking the dynamic trajectory of functional network changes and their association with tau distribution in EOAD patients with different clinical phenotypes across the preclinical to dementia stages of the disease will facilitate the development of accurate predictive models for EOAD.

### 3.3 Diffusion tensor image analysis along the perivascular space (DTI-ALPS)

Glymphatic system dysfunction, which leads to the accumulation of pathological proteins in the brain, is considered one of the potential pathogenic mechanisms of AD. The DTI-ALPS index is a novel quantitative marker for non-invasive assessment of the clearance function of the cerebral glymphatic system, calculated by the ratio of diffusivity perpendicular to the main fiber direction in specific white matter regions (projection fibers and association fibers) (66).

Existing studies have shown that the ALPS index is already abnormal before CSF Aβ42 reaches the positive threshold, and accelerates to decline after Aβ deposition (67). A retrospective cohort study by Yan et al. showed no significant difference in the ALPS index between the EOAD and LOAD groups. However, a significant correlation between the ALPS index and cognitive impairment (assessed by MMSE) was observed in the EOAD group, but not in the LOAD group. Furthermore, there was no significant correlation between the ALPS index and Aβ burden in the EOAD group (68), suggesting that glymphatic dysfunction in EOAD may involve factors other than Aβ plaque deposition, which remains to be further investigated.

Notably, a recent study demonstrated that the conventional ALPS index is significantly affected by the microstructural asymmetry of white matter fibers itself, which is associated with Aβ burden and age. After correction for this microstructural bias, the correlation between the ALPS index and Aβ burden disappeared, and its correlation with cognitive decline was also reduced (69). This finding indicates that the influence of white matter microstructure itself needs to be considered when interpreting the DTI-ALPS index.

### 3.4 Arterial spin labeling (ASL)

**Figure 1:**
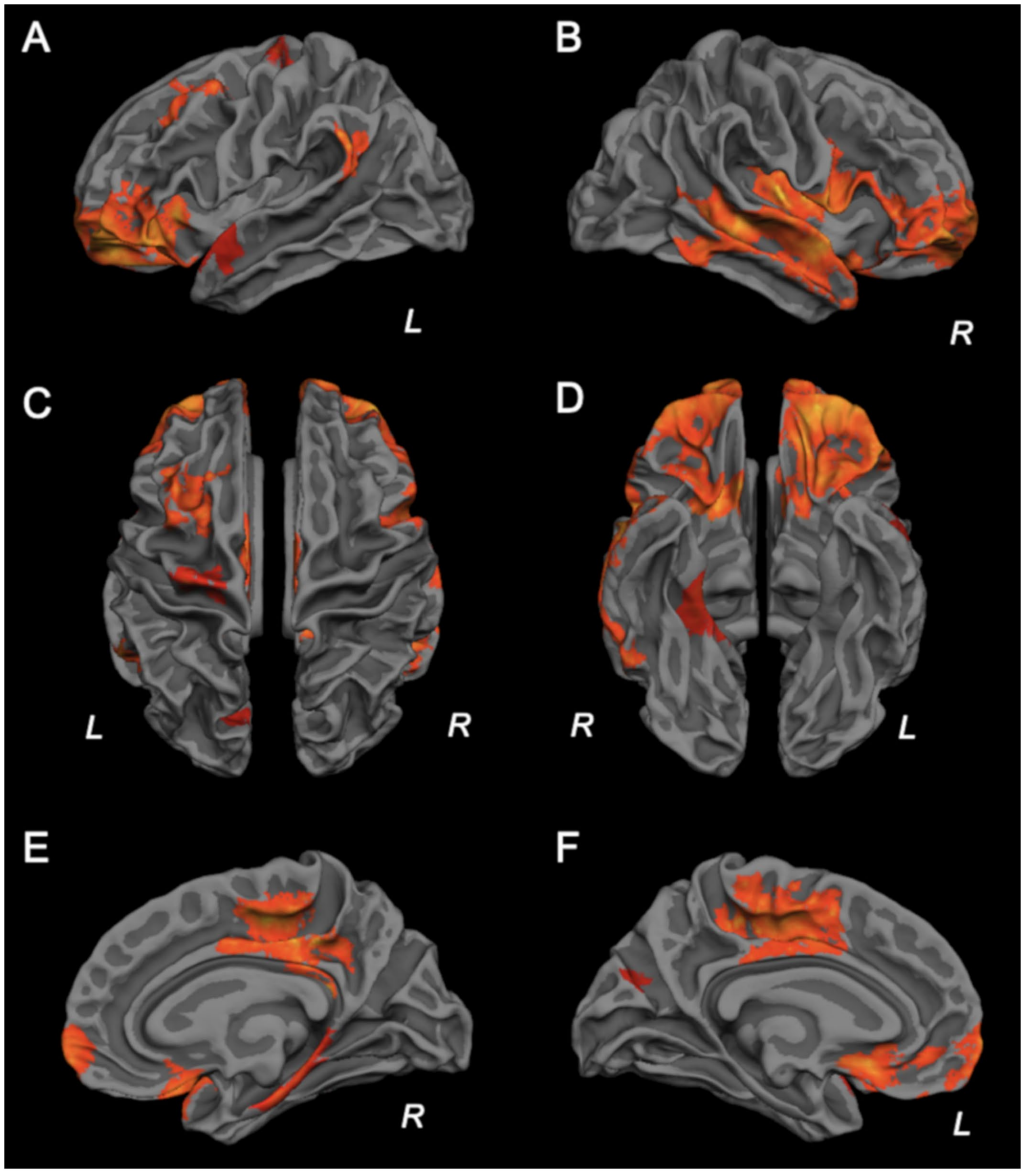
Cortical surface display showing the trend towards significant hypoperfusion in EOAD patients compared to LOAD patients with left lateral **(A)**, right lateral **(B)**, superior **(C)**, inferior **(D)**, right medial **(E)**, and left medial **(F)** views. Orange color represents areas of hypoperfusion in the EOAD patients located in PCC and precuneus, temporo-parietal regions and left superior and middle frontal gyrus. Italic R and L letters indicate the right and left sides. With permission from Verclytte et al. (70).

**Figure 2:**
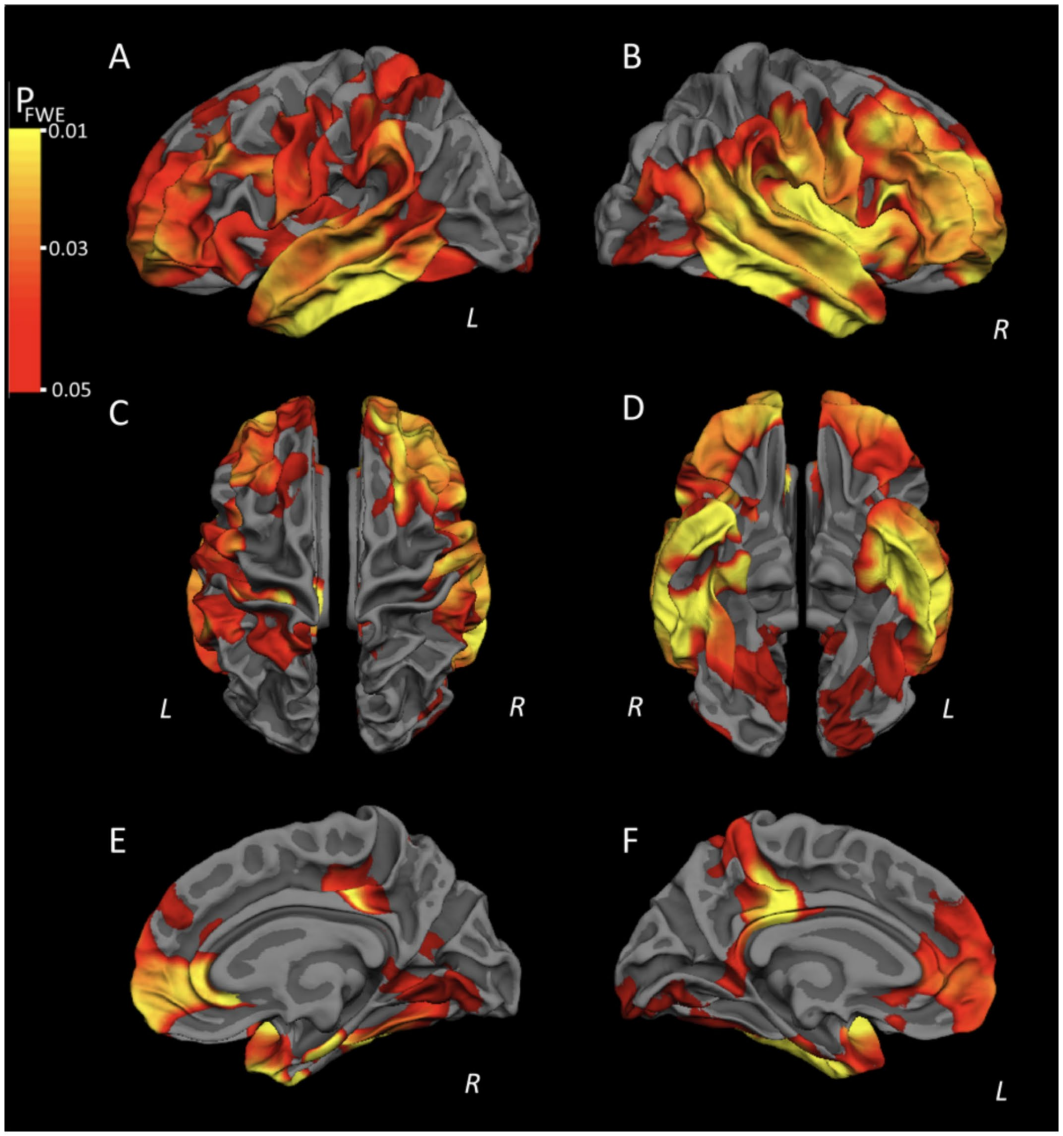
Significant areas of hypoperfusion in non-amnestic EOAD patients compared to amnestic patients plotting on cortical surfaces with left lateral **(A)**, right lateral **(B)**, superior **(C)**, inferior **(D)**, right medial **(E)**, and left medial **(F)** views. Warm colors (yellow and red) represent the areas of significant hypoperfusion in the non-amnestic EOAD patients (*p* < 0.05 FWE-corrected) located in the temporo-parietal neocortex, precuneus, PCC and frontal lobes. With permission from Verclytte et al. (70).

ASL is a non-invasive MRI technique that does not require contrast agent injection, and can quantify absolute cerebral blood flow (CBF). Verclytte et al. systematically compared cortical perfusion differences between EOAD patients with different clinical phenotypes, as well as between EOAD and LOAD, using ASL (70). The study showed that both amnestic and non-amnestic EOAD patients exhibited extensive hypoperfusion regions compared with healthy controls. Compared with amnestic EOAD, non-amnestic EOAD patients had lower perfusion in the bilateral temporoparietal lobes, precuneus, and anterior cingulate gyrus. Compared with the LOAD group, the EOAD group showed more severe hypoperfusion in the bilateral superior temporal gyri, bilateral frontal lobes, right anterior cingulate gyrus, and right precuneus (Figures 1, 2). However, the sample size of each group in this study was small, and the perfusion patterns of different EOAD clinical subtypes need to be verified in larger samples in the future.

Neurovascular coupling (NVC) reflects the interaction between local cerebral perfusion and neuronal activity within specific brain regions or functional networks. Combined BOLD and ASL techniques have been applied to NVC research, by calculating the correlation and ratio to reflect the synergy between cerebral perfusion and neuronal activity in each brain voxel (71). Longitudinal studies investigating the association between BOLD-ASL coupling, pathological proteins, and cognitive decline in the early stages of EOAD may help evaluate the diagnostic efficacy of BOLD-ASL coupling in the disease.

## 4 Positron emission tomography (PET)

PET techniques enable *in vivo* visualization of molecular pathological alterations, neuronal metabolism, neuroinflammation, synaptic density, and other core biological processes in the brain using specific tracers. They are critical tools for deciphering the pathological mechanisms of EOAD and exploring disease-specific biomarkers.

### 4.1 Aβ-PET and tau-PET

Increasing evidence indicates a spatiotemporal dissociation between Aβ plaques and tau protein during the course of AD. These two pathological proteins act synergistically in certain brain regions, while independently exerting effects in other regions to induce downstream neurodegenerative changes (72–74).

Some Aβ-PET studies have shown no significant difference in global Aβ burden between EOAD and LOAD (75, 76). However, other studies have reported higher Aβ burden in EOAD compared with LOAD (77–79). This inconsistency may be related to clinical differences in the study samples (APOE genotype, clinical phenotype), methodological differences (tracer type, partial volume correction, spatial normalization), and statistical power across studies.

**Figure 3:**
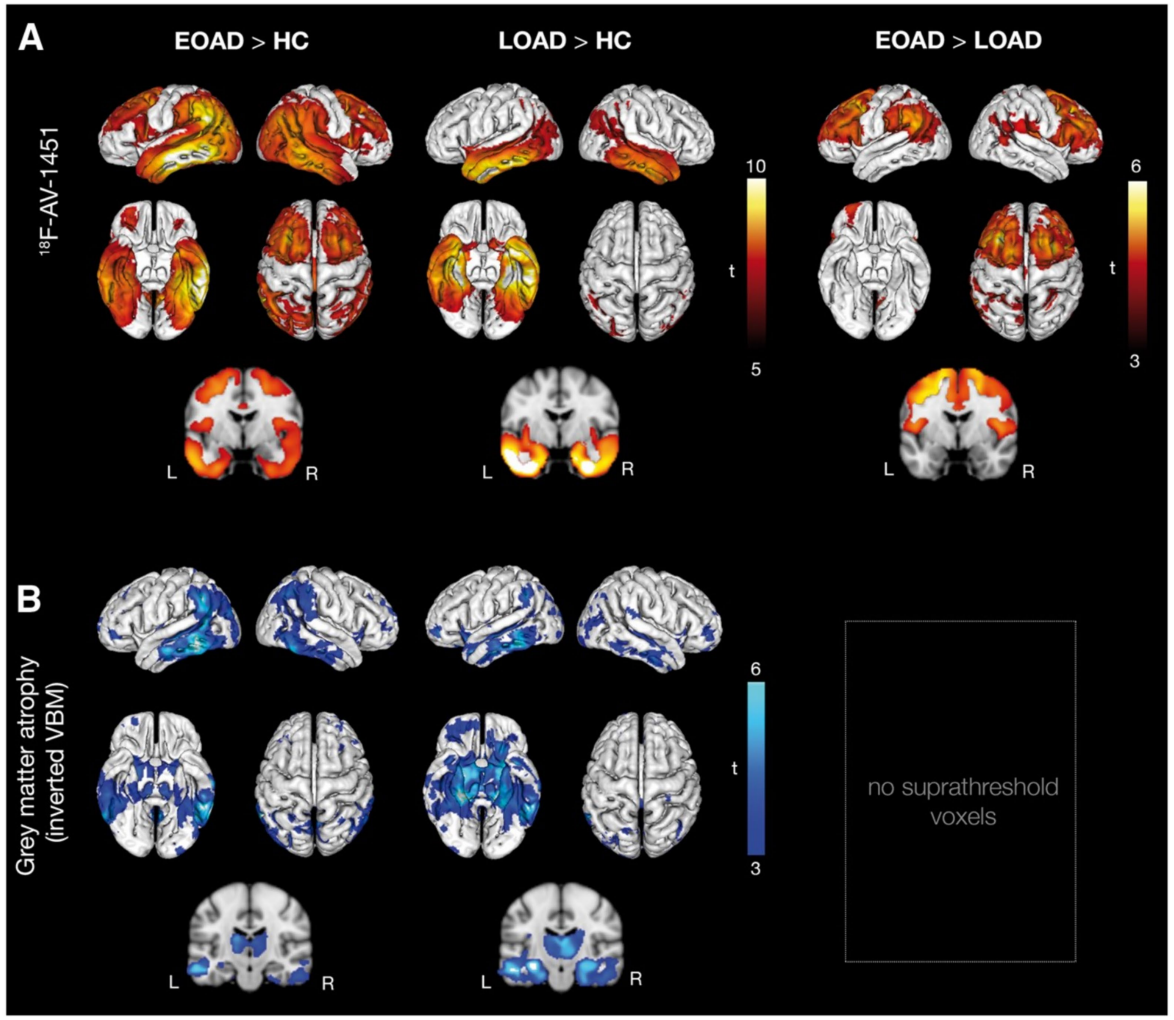
Group differences in ^18^F-AV-1451 and cortical atrophy. *T*-maps showing the results of voxelwise ANCOVA and two-sample *t*-test analyses. **(A)** Showing higher ^18^F-AV-1451 uptake in early-onset Alzheimer’s disease (EOAD) subjects when compared to healthy controls (HC), higher AV-1451 uptake in late-onset Alzheimer’s disease (LOAD) subjects when compared to healthy controls (ANCOVA; both *p* < 0.05, FWE), and higher ^18^F-AV-1451 uptake in patients with early-onset Alzheimer’s disease when compared to late-onset Alzheimer’s disease patients (*t*-test) (*p* < 0.001, uncorrected). **(B)** Greater grey matter atrophy in early-onset Alzheimer’s disease and late-onset Alzheimer’s disease patients when compared with healthy controls (ANCOVA; *p* < 0.001 uncorrected). No voxels survived statistically at this threshold when comparing early-onset Alzheimer’s disease directly to late-onset Alzheimer’s disease. VBM = voxel-based morphometry. With permission from Schöll et al. (13).

Existing tau-PET studies have found that LOAD patients have higher tau burden in the basal forebrain and medial temporal lobe (Figure 3), while EOAD patients show higher tau uptake in neocortical regions, including the precuneus, inferior parietal lobule, and dorsolateral prefrontal cortex (13, 18, 19, 80). Patients with PCA have high tau-PET signal in the occipital and parietal cortex, while patients with lvPPA have a higher burden in the left temporo-parietal areas (20, 81–83). In addition, the association between tau pathology in the lateral temporal and occipitoparietal lobes and cognitive impairment is stronger in EOAD than in LOAD (84).

In correlation analyses between Aβ-PET and tau-PET, LOAD patients showed a significant positive correlation between tau standardized uptake value ratio (SUVR) in the parietal and temporal lobes and Aβ SUVR across the entire neocortex. In contrast, EOAD patients only showed weak local correlations between tau SUVR and Aβ SUVR in 7 brain regions, with no correlation observed in distant brain regions (84). A mediation effect analysis based on the LEADS cohort by Cho et al. showed that the effect of Aβ on cognitive scores (MMSE, Montreal Cognitive Assessment [MoCA], Clinical Dementia Rating Scale Sum of Boxes [CDR-SB]) was fully mediated by tau protein (85).

The differences in pathological protein deposition patterns between EOAD and LOAD are critical for the development of treatment strategies for these patient populations. Multiple clinical trials of anti-Aβ monoclonal antibodies have shown that significant reduction of Aβ plaques (detected by Aβ-PET) is associated with reduced concentrations of phosphorylated tau protein in plasma and CSF, slowed accumulation of neurofibrillary tangles (detected by tau-PET), and delayed progression of cognitive decline (18, 86). Currently, there are limited published data on the efficacy of anti-Aβ drugs in EOAD, but a recent exploratory analysis of the Lecanemab trial suggested that younger patients may have a poorer treatment response than older patients (87). This observational finding needs to be further explored in clinical trials specifically designed for EOAD and LOAD cohorts.

The spatial distribution patterns and interaction between cerebral Aβ and tau protein in EOAD remain unclear. The affinity and selectivity of different tracers for Aβ and tau vary, and most existing studies are cross-sectional designs, which limit the direct comparison of results across studies. Future studies need to perform longitudinal follow-up with standardized image acquisition and processing methods to clarify the spatiotemporal relationship between Aβ and tau in EOAD.

### 4.2 [^18^F]-FDG PET

[^18^F]-fluorodeoxyglucose ([^18^F]-FDG) PET indirectly reflects synaptic activity of neurons by quantifying the glucose uptake rate in brain regions.

Existing studies have shown that EOAD patients have lower cerebral glucose metabolism than LOAD patients, particularly with significant hypometabolism in the parietal cortex, which is more prominent in the left cerebral hemisphere (75, 77, 88–92). Even in patients with amnestic EOAD, reduced metabolism in the left precuneus and left supramarginal gyrus was observed compared with LOAD (93).

**Figure 4:**
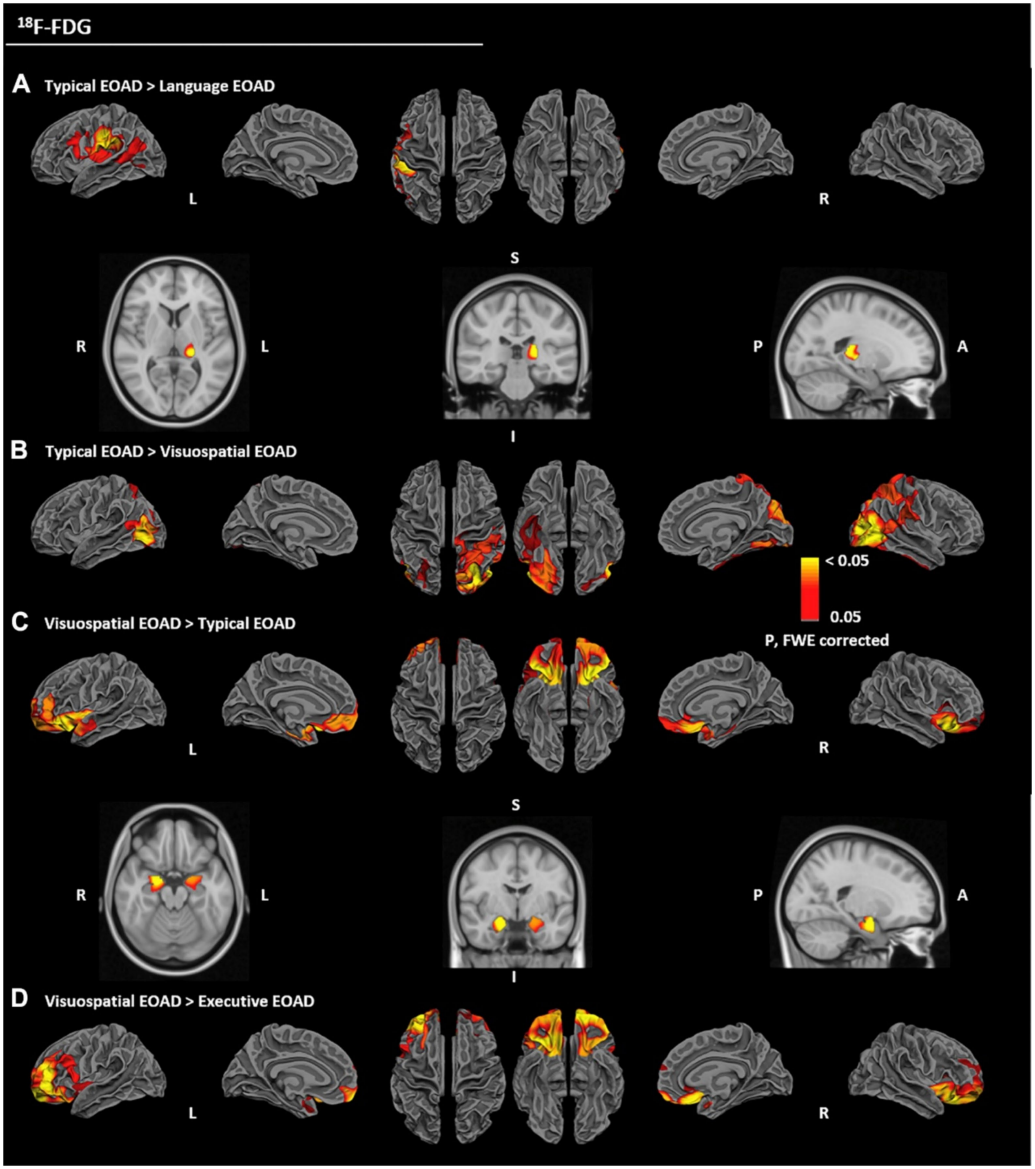
Hypometabolism patterns in typical versus atypical subgroups and visuospatial versus executive forms of early-onset Alzheimer’s disease (EOAD). Hypometabolic areas in language forms **(A)** and visuospatial forms **(B)**, relative to typical presentations of EOAD. Hypometabolism patterns in typical forms **(C)** and executive forms **(D)** relative to visuospatial presentations of EOAD. Statistical analyses were performed on common cortical surfaces (with regression of the cortical thickness; A-top row, B, C, and D-top row) and in MNI volume space for subcortical structures of interest (with regression of the gray matter volume; A-bottom row and D-bottom row). The color bar indicates the level of statistical significance, from red (least significant) to yellow (most significant). All clusters shown have a *p*-value <0.05 after familywise error correction. MNI, Montreal Neurological Institute. (For interpretation of the references to color in this figure legend, the reader is referred to the web version of this article) (94).

Vanhoutte et al. were the first to systematically compare the whole-brain metabolic patterns of different clinical subtypes of sporadic EOAD, after controlling for the effects of cortical atrophy (94). The language impairment subtype showed hypometabolism in Wernicke’s area, Broca’s area, insula, and pulvinar of the thalamus in the left cerebral hemisphere; the visuospatial impairment subtype exhibited hypometabolism in the bilateral parieto-occipital lobes, fusiform gyrus, and cuneus; the executive dysfunction subtype showed hypometabolism in the bilateral orbitofrontal cortex, dorsolateral and ventrolateral prefrontal cortex; and the typical amnestic subtype was characterized by hypometabolism mainly in the limbic system (Figure 4). In addition, the metabolic pattern of each subtype was linearly correlated with neuropsychological test scores of the corresponding cognitive domain, suggesting that the clinical heterogeneity of EOAD may be related to the differential susceptibility of distinct functional brain regions to the disease.

A study by Alice et al. found that EOAD patients with different CSF biomarker profiles exhibited distinct cerebral metabolic patterns. Elevated CSF tau levels were associated with hypometabolism in the bilateral frontal lobes and anterior cingulate gyrus, and the most prominent frontal hypometabolism was observed in the group with extremely high CSF tau levels (95).

Regional cerebral glucose metabolism is an early and progressive characteristic of AD. However, the relationship between cerebral metabolic changes and other pathological processes during the course of EOAD (such as Aβ plaque deposition, neurofibrillary tangle formation, neuroinflammation, and synaptic dysfunction) still needs to be further explored through integrated multimodal imaging.

### 4.3 TSPO-PET

Neuroinflammation mediated by microglial activation is recognized as one of the early pathological changes in AD (96–99). Existing studies have shown that microglial activity is higher in EOAD patients than in LOAD patients (100). The 18 kDa translocator protein (TSPO) is the most common target for inflammatory PET imaging, as it reflects the density of microglia in brain regions (101, 102).

A study by Tondo et al. combining ^18^F-FDG PET and [^11^C]-(R)-PK11195 PET (TSPO-PET) found that regions of reduced glucose metabolism in the inferior temporal gyrus, precuneus, angular gyrus, and inferior parietal lobule showed spatial overlap and a significant positive correlation with regions of dense microglial activation. Network analysis revealed a loss of long-range connectivity between this overlapping region and the frontal lobe, which was preserved in healthy controls (103). In addition, existing studies consistently found that neuroinflammation in sporadic EOAD is preferentially distributed along brain regions with strong functional connectivity, showing a certain spatial overlap with tau-PET imaging. However, the mechanism by which tau protein and neuroinflammation contribute to disease progression remains unclear (104).

It is important to note that the binding affinity of TSPO tracers is affected by the rs6971 polymorphism of the TSPO gene, which results in three binding phenotypes: high-affinity binder (HAB), medium-affinity binder (MAB), and low-affinity binder (LAB) (105). LAB individuals have low sensitivity to conventional TSPO tracers, which may introduce bias into studies. Appleton et al. used the next-generation tracer [^11^C]ER176 to achieve neuroinflammation imaging across all TSPO genotypes in EOAD for the first time, and found that the distribution of inflammation was more strongly correlated with tau deposition than with Aβ in EOAD patients at the mild cognitive impairment stage (106). Next-generation TSPO tracers are expected to provide more reliable tools for EOAD research.

TSPO-PET provides a unique perspective for *in vivo* studies of neuroinflammation in EOAD. However, it should be noted that upregulated TSPO expression is not a completely specific marker of microglial activation, and the results should be interpreted with caution. In addition, most current studies are cross-sectional, small-sample designs, and the conclusions need to be replicated and validated.

### 4.4 SV2A-PET

Synaptic loss is also one of the early pathological changes in AD (107, 108), and its correlation with cognitive decline is stronger than that of Aβ or tau burden (109–112). Synaptic vesicle glycoprotein 2A (SV2A) is a membrane protein expressed in synaptic vesicles of presynaptic axon terminals. [^11^C]-UCB-J is the most widely used SV2A-PET tracer in current research, for *in vivo* assessment of synaptic density.

One study showed that EOAD patients and frontotemporal dementia patients with GRN mutations (FTLD-GRN) had more significant reductions in synaptic density compared with LOAD patients (113).

Current SV2A-PET research on EOAD is still in its infancy. Whether the binding of SV2A tracers to SV2A reflects neuronal loss, reduced synaptic vesicles, or off-target binding to other pathological proteins still lacks neuropathological validation, which limits the interpretation of SV2A-PET imaging results. In addition, it remains unclear whether the quantitative relationship between SV2A density and synaptic number is consistent across different brain regions and pathological stages in EOAD patients. Future studies with standardized methods and multicenter longitudinal designs will help us understand the pathological mechanisms of EOAD.

## 5 Summary and outlook

This paper systematically reviews the research progress in multimodal neuroimaging of sporadic EOAD. Based on the existing evidence, the following robust findings have been identified regarding the neuroimaging characteristics of EOAD: (1) On structural imaging, compared with LOAD, EOAD shows more significant atrophy in the posterior cortex (precuneus, inferior parietal lobule) and more severe damage in the posterior cerebral white matter; (2) On functional imaging, EOAD is mainly characterized by reduced functional connectivity in the fronto-parietal networks; (3) On molecular and metabolic imaging, EOAD exhibits more severe and extensive tau burden and reduced cerebral glucose metabolism compared with LOAD. In addition, EOAD with different clinical phenotypes shows relatively characteristic differences in atrophy, metabolism, and network connectivity patterns, with a consistent spatial correspondence to clinical manifestations.

Although existing studies have initially outlined the framework of neuroimaging features of sporadic EOAD, there are still many key unresolved issues and limitations in current research. For example, what are the differences in pathological spreading pathways between sporadic EOAD and LOAD? Does tau-PET show alterations in the preclinical stage of sporadic EOAD? With the advancement of multimodal MRI and PET imaging, what role do fluid biomarkers play in disease diagnosis? How can they be integrated with neuroimaging to improve the sensitivity and specificity of disease diagnosis?

Furthermore, the vast majority of existing studies are cross-sectional designs, which can only identify correlations between imaging abnormalities, pathological changes, and cognitive impairment, but cannot clarify the causal relationship and temporal sequence of these alterations, lacking validation from long-term longitudinal studies. The neuroimaging characteristics and laterality differences of different clinical phenotypes still need to be further validated in large-sample studies with strict stratification by phenotype. Meanwhile, there is substantial heterogeneity in image acquisition parameters, post-processing methods, and quantitative standards across different studies, which directly leads to poor comparability and reproducibility of results across studies. Many imaging features identified in existing studies have not been systematically evaluated for their practical efficacy in early disease identification and differential diagnosis at the individual level, nor has their specificity for differentiating EOAD from other early-onset dementias such as frontotemporal lobar degeneration and dementia with Lewy bodies been clarified. As no universally accepted standardized acquisition and interpretation protocols for PET imaging have been established to date, we herein summarize the methodological characteristics of the core published PET studies in this field (Table 1).

**Table 1:** Methodological characteristics of core included PET studies.

Author & Year	Sample size (EOAD/LOAD/Control)	PET modality	Tracer type	PVE correction	Spatial normalization template
Touroutoglou et al. (2023) (26)	45/0/30	Aβ-PET, tau-PET	^18^F-Florbetapir, ^18^F-AV-1451	Yes	MNI152
Schöll et al. (2017) (13)	30/39/40	tau-PET	^18^F-AV-1451	Yes	MNI152
Ossenkoppele et al. (2012) (77)	22/25/20	Aβ-PET, FDG-PET	^11^C-PiB, ^18^F-FDG	No	SPM8 MNI
Cho et al. (2023) (76)	164/0/109	Aβ-PET, tau-PET	^18^F-Florbetapir, ^18^F-Flortaucipir	Yes	MNI305
Tondo et al. (2020) (103)	18/20/15	TSPO-PET, FDG-PET	[^11^C]-(R)-PK11195, ^18^F-FDG	Yes	MNI152
Appleton et al. (2025) (106)	25/0/20	TSPO-PET, Aβ-PET, tau-PET	[^11^C]ER176, ^18^F-Florbetaben, ^18^F-AV-1451	Yes	MNI305
Vanhoutte et al. (2017) (94)	32/0/28	FDG-PET	^18^F-FDG	Yes	SPM12 MNI
Na et al. (2024) (84)	36/42/30	Aβ-PET, tau-PET	^18^F-Florbetaben, ^18^F-Flortaucipir	Yes	MNI152

Future neuroimaging research on sporadic EOAD should focus on two key directions. On the one hand, large-sample, multicenter, long-term follow-up longitudinal cohort studies (such as LEADS) are needed, with strict stratification of patients by clinical phenotype, to systematically track the dynamic trajectory of multimodal imaging changes from the preclinical stage to the dementia stage of the disease, and clarify the temporal relationship between imaging abnormalities, pathological progression, and cognitive decline. On the other hand, it is necessary to promote the deep integration of multimodal imaging with genomics, fluid biomarkers, and cognitive phenotypes, to construct a multi-dimensional feature system of the disease, and deeply decipher its underlying pathophysiological mechanisms. These efforts will provide reliable imaging tools for the early identification, precise subtyping, individualized treatment, and efficacy monitoring of sporadic EOAD.

## References

[1] Zhu X-C Tan L Wang HF Jiang T Cao L Wang C . Rate of early onset Alzheimer’s disease: a systematic review and meta-analysis. Ann Transl Med. (2015) 3:38. doi: 10.3978/j.issn.2305-5839.2015.01.19, 25815299 PMC4356853

[2] Dw S Lw B Tp J R LJ Js Y. Dissecting the clinical heterogeneity of early-onset Alzheimer’s disease. Mol Psychiatry. (2022) 27:2674–88. doi: 10.1038/s41380-022-01531-9, 35393555 PMC9156414

[3] Zhang Z Han S Zhu H Wang Q Cheng S Han Y . Global, regional, and National Burden of early-onset Alzheimer’s disease and other dementias in young adults aged 40-64 years, 1990-2021: a population-based study. Eur J Neurol. (2025) 32:e70116. doi: 10.1111/ene.70116, 40105289 PMC11921012

[4] van Vliet D de Vugt ME Bakker C Pijnenburg YAL Vernooij-Dassen MJFJ Koopmans RTCM . Time to diagnosis in young-onset dementia as compared with late-onset dementia. Psychol Med. (2013) 43:423–32. doi: 10.1017/S0033291712001122, 22640548

[5] Mendez MF. Early-onset Alzheimer disease and its variants. Continuum. (2019) 25:34–51. doi: 10.1212/con.0000000000000687, 30707186 PMC6538053

[6] Heyman A Wilkinson WE Hurwitz BJ Helms MJ Haynes CS Utley CM . Early-onset Alzheimer’s disease: clinical predictors of institutionalization and death. Neurology. (1987) 37:980–4. doi: 10.1212/wnl.37.6.980 3587649

[7] Barclay LL Zemcov A Blass JP McDowell FH. Factors associated with duration of survival in Alzheimer’s disease. Biol Psychiatry. (1985) 20:86–93. doi: 10.1016/0006-3223(85)90139-8, 3965040

[8] Apostolova LG Aisen P Eloyan A Fagan A Fargo KN Foroud T . The longitudinal early-onset Alzheimer’s disease study (LEADS): framework and methodology. Alzheimers Dement. (2021) 17:2043–55. doi: 10.1002/alz.12350, 34018654 PMC8939858

[9] Koss E Edland S Fillenbaum G Mohs R Clark C Galasko D . Clinical and neuropsychological differences between patients with earlier and later onset of Alzheimer’s disease: a CERAD analysis, part XII. Neurology. (1996) 46:136–41. doi: 10.1212/wnl.46.1.136, 8559362

[10] Murray ME Graff-Radford NR Ross OA Petersen RC Duara R Dickson DW. Neuropathologically defined subtypes of Alzheimer’s disease with distinct clinical characteristics: a retrospective study. Lancet Neurol. (2011) 10:785–96. doi: 10.1016/S1474-4422(11)70156-9, 21802369 PMC3175379

[11] Mendez MF. Early-onset Alzheimer’s disease. Neurol Clin. (2017) 35:263–81. doi: 10.1016/j.ncl.2017.01.005, 28410659 PMC5407192

[12] Wu J Wang J Xiao Z Lu J Ma X Zhou X . Clinical characteristics and biomarker profile in early- and late-onset Alzheimer’s disease: the Shanghai memory study. Brain Commun. (2025) 7:fcaf015. doi: 10.1093/braincomms/fcaf015 39850631 PMC11756380

[13] Schöll M Ossenkoppele R Strandberg O Palmqvist S The Swedish BioFINDER study Jögi J . Distinct ^18^F-AV-1451 tau PET retention patterns in early- and late-onset Alzheimer’s disease. Brain J Neurol. (2017) 140:2286–94. doi: 10.1093/brain/awx171, 29050382

[14] Cho H Jeon S Kang SJ Lee JM Lee JH Kim GH . Longitudinal changes of cortical thickness in early- versus late-onset Alzheimer’s disease. Neurobiol Aging. (2013) 34:1921.e9–1921.e15. doi: 10.1016/j.neurobiolaging.2013.01.004, 23391426

[15] La Joie R Visani AV Baker SL Brown JA Bourakova V Cha J . Prospective longitudinal atrophy in Alzheimer’s disease correlates with the intensity and topography of baseline tau-PET. Sci Transl Med. (2020) 12:eaau5732. doi: 10.1126/scitranslmed.aau5732, 31894103 PMC7035952

[16] Spina S La Joie R Petersen C Nolan AL Cuevas D Cosme C . Comorbid neuropathological diagnoses in early versus late-onset Alzheimer’s disease. Brain. (2021) 144:2186–98. doi: 10.1093/brain/awab099, 33693619 PMC8502474

[17] Marshall GA Fairbanks LA Tekin S Vinters HV Cummings JL. Early-onset Alzheimer’s disease is associated with greater pathologic burden. J Geriatr Psychiatry Neurol. (2007) 20:29–33. doi: 10.1177/0891988706297086, 17341768

[18] Pontecorvo MJ Devous MD Navitsky M Lu M Salloway S Schaerf FW . Relationships between flortaucipir PET tau binding and amyloid burden, clinical diagnosis, age and cognition. Brain. (2017) 140:748–63. doi: 10.1093/brain/aww334, 28077397 PMC5382945

[19] Stage EC Svaldi D Phillips M Canela VH Duran T Goukasian N . Neurodegenerative changes in early- and late-onset cognitive impairment with and without brain amyloidosis. Alzheimer's Res Ther. (2020) 12:93. doi: 10.1186/s13195-020-00647-w, 32758274 PMC7409508

[20] La Joie R Visani AV Lesman-Segev OH Baker SL Edwards L Iaccarino L . Association of APOE4 and clinical variability in Alzheimer disease with the pattern of tau- and amyloid-PET. Neurology. (2021) 96:e650–61. doi: 10.1212/WNL.0000000000011270, 33262228 PMC7884991

[21] Dickerson BC Stoub TR Shah RC Sperling RA Killiany RJ Albert MS . Alzheimer-signature MRI biomarker predicts AD dementia in cognitively normal adults. Neurology. (2011) 76:1395–402. doi: 10.1212/WNL.0b013e3182166e96, 21490323 PMC3087406

[22] Dickerson BC Wolk DA Alzheimer’s Disease Neuroimaging Initiative. Biomarker-based prediction of progression in MCI: comparison of AD signature and hippocampal volume with spinal fluid amyloid-β and tau. Front Aging Neurosci. (2013) 5:55. doi: 10.3389/fnagi.2013.00055, 24130528 PMC3795312

[23] Dickerson BC Bakkour A Salat DH Feczko E Pacheco J Greve DN . The cortical signature of Alzheimer’s disease: regionally specific cortical thinning relates to symptom severity in very mild to mild AD dementia and is detectable in asymptomatic amyloid-positive individuals. Cereb. Cortex. (2009) 199:497–510. doi: 10.1093/cercor/bhn113 PMC2638813 18632739

[24] Racine AM Brickhouse M Wolk DA Dickerson BC. & Alzheimer’s Disease Neuroimaging Initiative. The personalized Alzheimer’s disease cortical thickness index predicts likely pathology and clinical progression in mild cognitive impairment. Alzheim Dem Amst Neth. (2018) 10:301–10. doi: 10.1016/j.dadm.2018.02.007, 29780874 PMC5956936

[25] C R E R Gw B. Late-onset vs nonmendelian early-onset Alzheimer disease: a distinction without a difference? Neurol Genet. (2020) 6:e512. doi: 10.1212/NXG.0000000000000512 33225065 PMC7673282

[26] Touroutoglou A Katsumi Y Brickhouse M Zaitsev A Eckbo R Aisen P . The sporadic early-onset Alzheimer’s disease signature of atrophy: preliminary findings from the longitudinal early-onset Alzheimer’s disease study (LEADS) cohort. Alzheimers Dement. (2023) 19:S74–88. doi: 10.1002/alz.13466 37850549 PMC10829523

[27] Mehta RI Keith CM CVL T Worhunsky PD Phelps HE Ward M . The early-onset Alzheimer’s disease MRI signature: a replication and extension analysis in early-stage AD. Cereb Cortex. (2024) 34:bhae475. doi: 10.1093/cercor/bhae475 39714256 PMC11664631

[28] Ossenkoppele R Cohn-Sheehy BI la Joie R Vogel JW Möller C Lehmann M . Atrophy patterns in early clinical stages across distinct phenotypes of Alzheimer’s disease. Hum Brain Mapp. (2015) 36:4421–37. doi: 10.1002/hbm.22927, 26260856 PMC4692964

[29] Montembeault M Brambati SM Gorno-Tempini ML Migliaccio R. Clinical, anatomical, and pathological features in the three variants of primary progressive aphasia: a review. Front Neurol. (2018) 9:692. doi: 10.3389/fneur.2018.00692, 30186225 PMC6110931

[30] Risacher SL Saykin AJ. Neuroimaging in aging and neurologic diseases. Handb Clin Neurol. (2019) 167:191–227. doi: 10.1016/B978-0-12-804766-8.00012-1, 31753134 PMC9006168

[31] Crutch SJ Lehmann M Schott JM Rabinovici GD Rossor MN Fox NC. Posterior cortical atrophy. Lancet Neurol. (2012) 11:170–8. doi: 10.1016/S1474-4422(11)70289-7, 22265212 PMC3740271

[32] Montembeault M Lacomblez L Habert M-O Kas A Migliaccio R. Posterior cortical atrophy: from vision to emotion. Geriatr Psychol Neuropsychiatr Vieil. (2018) 16:57–66. doi: 10.1684/pnv.2017.0717, 29569567

[33] Lv X Cheng Z Wang Q Gao F Dai L Du C . High burdens of phosphorylated tau protein and distinct precuneus atrophy in sporadic early-onset Alzheimer’s disease. Sci Bull. (2023) 68:2817–26. doi: 10.1016/j.scib.2023.10.019, 37919158

[34] Migliaccio R Agosta F Rascovsky K Karydas A Bonasera S Rabinovici GD . Clinical syndromes associated with posterior atrophy. Neurology. (2009) 73:1571–8. doi: 10.1212/WNL.0b013e3181c0d427, 19901249 PMC2777069

[35] Gour N Felician O Didic M Koric L Gueriot C Chanoine V . Functional connectivity changes differ in early and late-onset Alzheimer’s disease. Hum Brain Mapp. (2014) 35:2978–94. doi: 10.1002/hbm.22379, 24123475 PMC6869697

[36] Eloyan A Vemuri P Gatsonis C Carrillo MC Rabinovici GD Apostolova LG . White matter hyperintensities are higher among early-onset Alzheimer’s disease participants than their cognitively normal and early-onset nonAD peers: LEADS study. Alzheimers Dement. (2023) 19:S89–97. doi: 10.1002/alz.067918 37491599 PMC10808262

[37] Pham NTT Graff-Radford J Machulda MM Spychalla AJ Schwarz CG Senjem ML . Regional white matter hyperintensities in posterior cortical atrophy and logopenic progressive aphasia. Neurobiol Aging. (2022) 119:46–55. doi: 10.1016/j.neurobiolaging.2022.07.008, 35970009 PMC9886198

[38] Montembeault M Migliaccio R. Atypical forms of Alzheimer’s disease: patients not to forget. Curr Opin Neurol. (2023) 36:245–52. doi: 10.1097/wco.0000000000001182, 37365819

[39] Luo X Hong H Li K Zeng Q Wang S Li Z . Distinct cerebral small vessel disease impairment in early- and late-onset Alzheimer’s disease. Ann Clin Transl Neurol. (2023) 10:1326–37. doi: 10.1002/acn3.51824, 37345812 PMC10424647

[40] Li K-C Luo X Zeng QZ Xu XJ Huang PY Shen ZJ . Distinct patterns of interhemispheric connectivity in patients with early- and late-onset Alzheimer’s disease. Front Aging Neurosci. (2018) 10:261. doi: 10.3389/fnagi.2018.00261, 30237764 PMC6136638

[41] Galantucci S Tartaglia MC Wilson SM Henry ML Filippi M Agosta F . White matter damage in primary progressive aphasias: a diffusion tensor tractography study. Brain J Neurol. (2011) 134:3011–29. doi: 10.1093/brain/awr099, 21666264 PMC3187537

[42] Mahoney CJ Malone IB Ridgway GR Buckley AH Downey LE Golden HL . White matter tract signatures of the progressive aphasias. Neurobiol Aging. (2013) 34:1687–99. doi: 10.1016/j.neurobiolaging.2012.12.002, 23312804 PMC3601331

[43] Magnin E Sylvestre G Lenoir F Dariel E Bonnet L Chopard G . Logopenic syndrome in posterior cortical atrophy. J Neurol. (2013) 260:528–33. doi: 10.1007/s00415-012-6671-7 23007194

[44] Migliaccio R Agosta F Scola E Magnani G Cappa SF Pagani E . Ventral and dorsal visual streams in posterior cortical atrophy: a DT MRI study. Neurobiol Aging. (2012) 33:2572–84. doi: 10.1016/j.neurobiolaging.2011.12.025, 22277261 PMC4827710

[45] Migliaccio R Agosta F Toba MN Samri D Corlier F de Souza LC . Brain networks in posterior cortical atrophy: a single case tractography study and literature review. Cortex. (2012) 48:1298–309. doi: 10.1016/j.cortex.2011.10.002, 22099855 PMC4813795

[46] Kim M-J Seo SW Kim ST Lee J-M Na DL. Diffusion tensor changes according to age at onset and apolipoprotein E genotype in Alzheimer disease. Alzheimer Dis Assoc Disord. (2016) 30:297–304. doi: 10.1097/WAD.0000000000000155, 27227996

[47] Canu E Agosta F Spinelli EG Magnani G Marcone A Scola E . White matter microstructural damage in Alzheimer’s disease at different ages of onset. Neurobiol Aging. (2013) 34:2331–40. doi: 10.1016/j.neurobiolaging.2013.03.026, 23623599

[48] Caso F Agosta F Mattavelli D Migliaccio R Canu E Magnani G . White matter degeneration in atypical Alzheimer disease. Radiology. (2015) 277:162–72. doi: 10.1148/radiol.2015142766, 26018810

[49] Seeley WW Menon V Schatzberg AF Keller J Glover GH Kenna H . Dissociable intrinsic connectivity networks for salience processing and executive control. J Neurosci. (2007) 27:2349–56. doi: 10.1523/JNEUROSCI.5587-06.2007, 17329432 PMC2680293

[50] Smits LL Pijnenburg YA Koedam EL van der Vlies AE Reuling IE Koene T . Early onset Alzheimer’s disease is associated with a distinct neuropsychological profile. J Alzheimer's Dis. (2012) 30:101–8. doi: 10.3233/JAD-2012-111934 22366769

[51] Koedam ELGE Lauffer V van der Vlies AE van der Flier WM Scheltens P Pijnenburg YAL. Early-versus late-onset Alzheimer’s disease: more than age alone. J Alzheimer's Dis. (2010) 19:JAD19:1401–8. doi: 10.3233/JAD-2010-1337, 20061618

[52] Frisoni GB Pievani M Testa C Sabattoli F Bresciani L Bonetti M . The topography of grey matter involvement in early and late onset Alzheimer’s disease. Brain J Neurol. (2007) 130:720–30. doi: 10.1093/brain/awl377, 17293358

[53] Kalpouzos G Eustache F Sayette V Viader F Chételat G Desgranges B. Working memory and FDG-PET dissociate early and late onset Alzheimer disease patients. J Neurol. (2005) 252:548–58. doi: 10.1007/s00415-005-0685-3, 15726251

[54] Whitwell JL Jones DT Duffy JR Strand EA Machulda MM Przybelski SA . Working memory and language network dysfunctions in logopenic aphasia: a task-free fMRI comparison with Alzheimer’s dementia. Neurobiol Aging. (2015) 36:1245–52. doi: 10.1016/j.neurobiolaging.2014.12.013, 25592958 PMC4346438

[55] Hampel H. Amyloid-β and cognition in aging and Alzheimer’s disease: molecular and neurophysiological mechanisms. J Alzheimer's Dis. (2013) 33:JAD33:S79–86. doi: 10.3233/JAD-2012-129003 22531423

[56] de Haan W van der Flier WM Koene T Smits LL Scheltens P Stam CJ. Disrupted modular brain dynamics reflect cognitive dysfunction in Alzheimer’s disease. NeuroImage. (2012) 59:3085–93. doi: 10.1016/j.neuroimage.2011.11.055, 22154957

[57] Krajcovicova L Mikl M Marecek R Rektorova I. Disturbed default mode network connectivity patterns in Alzheimer’s disease associated with visual processing. J Alzheimer's Dis. (2014) 41:JAD41:1229–38. doi: 10.3233/JAD-131208, 24799341

[58] de Bruin H Groot C Barthel H Bischof GN Blazhenets G Boellaard R . Connectivity as a universal predictor of tau progression in atypical Alzheimer’s disease. Brain. (2025) 148:awaf279. doi: 10.1093/brain/awaf279, 40810361 PMC12588720

[59] Putcha D Brickhouse M Touroutoglou A Collins JA Quimby M Wong B . Visual cognition in non-amnestic Alzheimer’s disease: relations to tau, amyloid, and cortical atrophy. NeuroImage Clin. (2019) 23:101889. doi: 10.1016/j.nicl.2019.101889, 31200149 PMC6562373

[60] Putcha D Eckbo R Katsumi Y Dickerson BC Touroutoglou A Collins JA. Tau and the fractionated default mode network in atypical Alzheimer’s disease. Brain Commun. (2022) 4:fcac055. doi: 10.1093/braincomms/fcac055, 35356035 PMC8963312

[61] Katsumi Y Howe IA Eckbo R Wong B Quimby M Hochberg D . Default mode network tau predicts future clinical decline in atypical early Alzheimer’s disease. Brain. (2025) 148:1329–44. doi: 10.1093/brain/awae327, 39412999 PMC11969453

[62] de Calignon A Polydoro M Suárez-Calvet M William C Adamowicz DH Kopeikina KJ . Propagation of tau pathology in a model of early Alzheimer’s disease. Neuron. (2012) 73:685–97. doi: 10.1016/j.neuron.2011.11.033 22365544 PMC3292759

[63] Walker LC Marc D I Duff KE Hyman BT. Mechanisms of protein seeding in neurodegenerative diseases. JAMA Neurol. (2013) 70:304–10. doi: 10.1001/jamaneurol.2013.1453, 23599928 PMC3665718

[64] Kuchibhotla KV Wegmann S Kopeikina KJ Hawkes J Rudinskiy N Andermann ML . Neurofibrillary tangle-bearing neurons are functionally integrated in cortical circuits in vivo. Proc Natl Acad Sci USA. (2014) 111:510–4. doi: 10.1073/pnas.1318807111, 24368848 PMC3890777

[65] Yang F Chowdhury SR Jacobs HIL Sepulcre J Wedeen VJ Johnson KA . Longitudinal predictive modeling of tau progression along the structural connectome. NeuroImage. (2021) 237:118126. doi: 10.1016/j.neuroimage.2021.118126, 33957234 PMC8260445

[66] Taoka T Masutani Y Kawai H Nakane T Matsuoka K Yasuno F . Evaluation of glymphatic system activity with the diffusion MR technique: diffusion tensor image analysis along the perivascular space (DTI-ALPS) in Alzheimer’s disease cases. Jpn J Radiol. (2017) 35:172–8. doi: 10.1007/s11604-017-0617-z, 28197821

[67] Huang S-Y Zhang YR Guo Y du J Ren P Wu BS . Glymphatic system dysfunction predicts amyloid deposition, neurodegeneration, and clinical progression in Alzheimer’s disease. Alzheimers Dement. (2024) 20:3251–69. doi: 10.1002/alz.13789, 38501315 PMC11095446

[68] Zhang Y Huang G Geng J Li X Xin M Yuan P . DTI-ALPS index-assessed glymphatic dysfunction mediates Alzheimer’s cognitive decline via amyloid-β-dependent pathways: multimodal PET/MRI study. Eur J Nucl Med Mol Imaging. (2025) 53:467–79. doi: 10.1007/s00259-025-07445-2, 40679601

[69] Li S Chen R Cao Z Zhu Q Ma Y Zhu K . Microstructural Bias in the assessment of periventricular flow as revealed in postmortem brains. Radiology. (2025) 316:e250753. doi: 10.1148/radiol.250753, 40956166

[70] Verclytte S Lopes R Viard R Rollin A Vanhoutte M Pasquier F . Differences in cortical perfusion detected by arterial spin labeling in nonamnestic and amnestic subtypes of early-onset Alzheimer’s disease. J Neuroradiol. (2020) 47:284–91. doi: 10.1016/j.neurad.2019.03.017, 30981825

[71] Liang X Zou Q He Y Yang Y. Coupling of functional connectivity and regional cerebral blood flow reveals a physiological basis for network hubs of the human brain. Proc Natl Acad Sci USA. (2013) 110:1929–34. doi: 10.1073/pnas.1214900110, 23319644 PMC3562840

[72] Sepulcre J Schultz AP Sabuncu M Gomez-Isla T Chhatwal J Becker A . In vivo tau, amyloid, and gray matter profiles in the aging brain. J Neurosci. (2016) 36:7364–74. doi: 10.1523/JNEUROSCI.0639-16.2016, 27413148 PMC4945661

[73] Pascoal TA Mathotaarachchi S Mohades S Benedet AL Chung CO Shin M . Amyloid-β and hyperphosphorylated tau synergy drives metabolic decline in preclinical Alzheimer’s disease. Mol Psychiatry. (2017) 22:306–11. doi: 10.1038/mp.2016.37, 27021814 PMC5262471

[74] Sw P Ra N Nk R J S Lh T. Amyloid-independent mechanisms in Alzheimer’s disease pathogenesis. J Neurosci. (2010) 30:14946–54. doi: 10.1523/JNEUROSCI.4305-10.2010 21068297 PMC3426835

[75] Rabinovici GD Furst AJ Alkalay A Racine CA O’Neil JP Janabi M . Increased metabolic vulnerability in early-onset Alzheimer’s disease is not related to amyloid burden. Brain. (2010) 133:512–28. doi: 10.1093/brain/awp326, 20080878 PMC2858015

[76] Cho H Seo SW Kim J-H Suh MK Lee J-H Choe YS . Amyloid deposition in early onset versus late onset Alzheimer’s disease. J Alzheimers Dis. (2013) 35:813–21. doi: 10.3233/jad-121927, 23507771

[77] Ossenkoppele R Zwan MD Tolboom N van Assema DME Adriaanse SF Kloet RW . Amyloid burden and metabolic function in early-onset Alzheimer’s disease: parietal lobe involvement. Brain J Neurol. (2012) 135:2115–25. doi: 10.1093/brain/aws113, 22556189

[78] Choo IH Lee DY Kim JW Seo EH Lee DS Kim YK . Relationship of amyloid-β burden with age-at-onset in Alzheimer disease. Am J Geriatr Psychiatry. (2011) 19:627–34. doi: 10.1097/JGP.0b013e318202bf3a 21709608

[79] Li J Antonecchia E Camerlenghi M Chiaravalloti A Chu Q Costanzo AD . Correlation of [^18^F]florbetaben textural features and age of onset of Alzheimer’s disease: a principal components analysis approach. EJNMMI Res. (2021) 11:40. doi: 10.1186/s13550-021-00774-x, 33881633 PMC8060386

[80] Tanner JA Iaccarino L Edwards L Asken BM Gorno-Tempini ML Kramer JH . Amyloid, tau and metabolic PET correlates of cognition in early and late-onset Alzheimer’s disease. Brain J Neurol. (2022) 145:4489–505. doi: 10.1093/brain/awac229, 35762829 PMC10200306

[81] Xia C Makaretz SJ Caso C McGinnis S Gomperts SN Sepulcre J . Association of in Vivo [^18^F]AV-1451 tau PET imaging results with cortical atrophy and symptoms in typical and atypical Alzheimer disease. JAMA Neurol. (2017) 74:427–36. doi: 10.1001/jamaneurol.2016.5755, 28241163 PMC5470368

[82] Nasrallah IM Chen YJ Hsieh MK Phillips JS Ternes K Stockbower GE . ^18^F-Flortaucipir PET/MRI correlations in nonamnestic and amnestic variants of Alzheimer disease. J Nucl Med. (2018) 59:299–306. doi: 10.2967/jnumed.117.194282, 28747523 PMC6348438

[83] Sintini I Schwarz CG Martin PR Graff-Radford J Machulda MM Senjem ML . Regional multimodal relationships between tau, hypometabolism, atrophy, and fractional anisotropy in atypical Alzheimer’s disease. Hum Brain Mapp. (2019) 40:1618–31. doi: 10.1002/hbm.24473, 30549156 PMC6615561

[84] Na HK Shin JH Kim SW Seo S Kim WR Kang JM . Diverging relationships among amyloid, tau, and brain atrophy in early-onset and late-onset Alzheimer’s disease. Yonsei Med J. (2024) 65:434–47. doi: 10.3349/ymj.2023.0308, 39048319 PMC11284308

[85] Cho H Mundada NS Apostolova LG Carrillo MC Shankar R Amuiri AN . Amyloid and tau-PET in early-onset AD: baseline data from the longitudinal early-onset Alzheimer’s disease study (LEADS). Alzheimers Dement. (2023) 19:S98–S114. doi: 10.1002/alz.13453 37690109 PMC10807231

[86] Pontecorvo MJ Lu M Burnham SC Schade AE Dage JL Shcherbinin S . Association of Donanemab Treatment with Exploratory Plasma Biomarkers in early symptomatic Alzheimer disease: a secondary analysis of the TRAILBLAZER-ALZ randomized clinical trial. JAMA Neurol. (2022) 79:1250–9. doi: 10.1001/jamaneurol.2022.3392, 36251300 PMC9577883

[87] van Dyck CH Swanson CJ Aisen P Bateman RJ Chen C Gee M . Lecanemab in early Alzheimer’s disease. N Engl J Med. (2023) 388:9–21. doi: 10.1056/NEJMoa2212948, 36449413

[88] Aziz A-L Giusiano B Joubert S Duprat L Didic M Gueriot C . Difference in imaging biomarkers of neurodegeneration between early and late-onset amnestic Alzheimer’s disease. Neurobiol Aging. (2017) 54:22–30. doi: 10.1016/j.neurobiolaging.2017.02.010, 28314160

[89] Joubert S Gour N Guedj E Didic M Guériot C Koric L . Early-onset and late-onset Alzheimer’s disease are associated with distinct patterns of memory impairment. Cortex. (2016) 74:217–32. doi: 10.1016/j.cortex.2015.10.014, 26694580

[90] Kaiser NC Melrose RJ Liu C Sultzer DL Jimenez E Su M . Neuropsychological and neuroimaging markers in early versus late-onset Alzheimer’s disease. Am J Alzheimers Dis Other Dement. (2012) 27:520–9. doi: 10.1177/1533317512459798, 22990206 PMC4112191

[91] Kim EJ Cho SS Jeong Y Park KC Kang SJ Kang E . Glucose metabolism in early onset versus late onset Alzheimer’s disease: an SPM analysis of 120 patients. Brain. (2005) 128:1790–801. doi: 10.1093/brain/awh539, 15888536

[92] Sakamoto S Ishii K Sasaki M Hosaka K Mori T Matsui M . Differences in cerebral metabolic impairment between early and late onset types of Alzheimer’s disease. J Neurol Sci. (2002) 200:27–32. doi: 10.1016/S0022-510X(02)00114-4, 12127672

[93] Chiaravalloti A Koch G Toniolo S Belli L di Lorenzo F Gaudenzi S . Comparison between early-onset and late-onset Alzheimer’s disease patients with amnestic presentation: CSF and (18)F-FDG PET study. Dement Geriatr Cogn Disord Extra. (2016) 6:108–19. doi: 10.1159/000441776, 27195000 PMC4868930

[94] Vanhoutte M Semah F Rollin Sillaire A Jaillard A Petyt G Kuchcinski G . ^18^F-FDG PET hypometabolism patterns reflect clinical heterogeneity in sporadic forms of early-onset Alzheimer’s disease. Neurobiol Aging. (2017) 59:184–96. doi: 10.1016/j.neurobiolaging.2017.08.009 28882421

[95] Jaillard A Vanhoutte M Maureille A Schraen S Skrobala E Delbeuck X . The relationship between CSF biomarkers and cerebral metabolism in early-onset Alzheimer’s disease. Eur J Nucl Med Mol Imaging. (2019) 46:324–33. doi: 10.1007/s00259-018-4113-1 30155553

[96] Yokokura M Mori N Yagi S Yoshikawa E Kikuchi M Yoshihara Y . In vivo changes in microglial activation and amyloid deposits in brain regions with hypometabolism in Alzheimer’s disease. Eur J Nucl Med Mol Imaging. (2011) 38:343–51. doi: 10.1007/s00259-010-1612-0, 20844871

[97] Fan Z Aman Y Ahmed I Chetelat G Landeau B Ray Chaudhuri K . Influence of microglial activation on neuronal function in Alzheimer’s and Parkinson’s disease dementia. Alzheimers Dement. (2015) 11:608–621.e7. doi: 10.1016/j.jalz.2014.06.016, 25239737

[98] Cagnin A Brooks DJ Kennedy AM Gunn RN Myers R Turkheimer FE . In-vivo measurement of activated microglia in dementia. Lancet Lond Engl. (2001) 358:461–7. doi: 10.1016/S0140-6736(01)05625-2, 11513911

[99] Edison P Archer HA Gerhard A Hinz R Pavese N Turkheimer FE . Microglia, amyloid, and cognition in Alzheimer’s disease: an [^11^C](R)PK11195-PET and [^11^C]PIB-PET study. Neurobiol Dis. (2008) 32:412–9. doi: 10.1016/j.nbd.2008.08.001, 18786637

[100] Kreisl WC Lyoo CH McGwier M Snow J Jenko KJ Kimura N . In vivo radioligand binding to translocator protein correlates with severity of Alzheimer’s disease. Brain J Neurol. (2013) 136:2228–38. doi: 10.1093/brain/awt145, 23775979 PMC3692038

[101] Turkheimer FE Rizzo G Bloomfield PS Howes O Zanotti-Fregonara P Bertoldo A . The methodology of TSPO imaging with positron emission tomography. Biochem Soc Trans. (2015) 43:586–92. doi: 10.1042/BST20150058, 26551697 PMC4613512

[102] Nutma E Fancy N Weinert M Tsartsalis S Marzin MC Muirhead RCJ . Translocator protein is a marker of activated microglia in rodent models but not human neurodegenerative diseases. Nat Commun. (2023) 14:5247. doi: 10.1038/s41467-023-40937-z, 37640701 PMC10462763

[103] Tondo G Iaccarino L Caminiti SP Presotto L Santangelo R Iannaccone S . The combined effects of microglia activation and brain glucose hypometabolism in early-onset Alzheimer’s disease. Alzheimer's Res Ther. (2020) 12:50. doi: 10.1186/s13195-020-00619-0 32354345 PMC7193377

[104] Rauchmann B-S Ersözlü E Luedecke D Franzmeier N Perneczky R. Multimodal and longitudinal characterization of distinct tau and atrophy clusters in Alzheimer’s disease spectrum. Sci Rep. (2025) 15:18142. doi: 10.1038/s41598-025-98338-9, 40415101 PMC12104337

[105] Owen DR Yeo AJ Gunn RN Song K Wadsworth G Lewis A . An 18-kDa translocator protein (TSPO) polymorphism explains differences in binding affinity of the PET radioligand PBR28. J Cereb Blood Flow Metab. (2012) 32:1–5. doi: 10.1038/jcbfm.2011.147, 22008728 PMC3323305

[106] Appleton J Finn Q Zanotti-Fregonara P Yu M Faridar A Nakawah MO . Brain inflammation co-localizes highly with tau in mild cognitive impairment due to early-onset Alzheimer’s disease. Brain. (2025) 148:119–32. doi: 10.1093/brain/awae234, 39013020 PMC11706285

[107] Selkoe DJ. Alzheimer’s disease is a synaptic failure. Science. (2002) 298:789–91. doi: 10.1126/science.1074069, 12399581

[108] Scheff SW DeKosky ST Price DA. Quantitative assessment of cortical synaptic density in Alzheimer’s disease. Neurobiol Aging. (1990) 11:29–37. doi: 10.1016/0197-4580(90)90059-9, 2325814

[109] Terry RD . Physical basis of cognitive alterations in Alzheimer’s disease: synapse loss is the major correlate of cognitive impairment. Ann Neurol. (1991) 30:572–80. doi: 10.1002/ana.410300410, 1789684

[110] DeKosky ST Scheff SW. Synapse loss in frontal cortex biopsies in Alzheimer’s disease: correlation with cognitive severity. Ann Neurol. (1990) 27:457–64. doi: 10.1002/ana.410270502, 2360787

[111] DeKosky ST Scheff SW Styren SD. Structural correlates of cognition in dementia: quantification and assessment of synapse change. Neurodegener. (1996) 5:417–21. doi: 10.1006/neur.1996.0056, 9117556

[112] Hamos JE DeGennaro LJ Drachman DA. Synaptic loss in Alzheimer’s disease and other dementias. Neurology. (1989) 39:355–61. doi: 10.1212/WNL.39.3.355 2927643

[113] Bavarsad MS Shanaki Bavarsad M Spina S Oehler A Allen IE Suemoto CK . Comprehensive mapping of synaptic vesicle protein 2A (SV2A) in health and neurodegenerative diseases: a comparative analysis with synaptophysin and ground truth for PET imaging interpretation. Acta Neuropathol. (2024) 148:58. doi: 10.1007/s00401-024-02816-9, 39476256 PMC11827533

